# Assessment of point-of-care creatinine testing in pregnancy: a prospective cohort study in Sierra Leone

**DOI:** 10.1016/j.lanafr.2026.100069

**Published:** 2026-08

**Authors:** Katy Kuhrt, Rossetta Cole, Moses M’Bayoh, Christiana Squire, Alhaji Kamara, Gabriel Ganyaglo, Joshua Coker, Katherine Clark, Alice Beardmore-Gray, Alexandra Ridout, Christina Fernandez-Turienzo, Jackie Campbell, Adetunji Adeniji, Lucy C. Chappell, Andrew H. Shennan, Kate Bramham

**Affiliations:** a Department of Women and Children’s Health, St Thomas’s Hospital, Westminster Bridge Road, London, SE1 7EH, UK; b Princess Christian Maternity Hospital, Ministry of Health and Sanitation, Fourah Bay Rd, Freetown, Sierra Leone; c Connaught Hospital, 1 Percival Street, Freetown, Sierra Leone; d Faculty of Health, Education and Society, University of Northampton, University Drive, Northampton, NN1 5PH, UK

**Keywords:** Pregnancy-associated AKI, Acute kidney injury, Adverse pregnancy-related outcomes

## Abstract

**Background:**

Pregnancy-associated acute kidney injury (PrAKI) is a major contributor to maternal and perinatal morbidity and mortality in low-and middle-income countries, where detection is often delayed due to limited access to creatinine testing. Point-of-care creatinine (POC-Cr) devices may offer a practical alternative. We aimed to describe the incidence, resolution, and clinical consequences of presumed PrAKI or kidney dysfunction among high-risk pregnant and postpartum women, and to assess feasibility of POC-Cr testing.

**Methods:**

We conducted a study of pregnant or postpartum women at high risk of PrAKI at a hospital in Freetown, Sierra Leone (April–August 2023). Eligibility criteria were systolic blood pressure ≥140 mmHg, diastolic ≥90 mmHg, and/or shock index ≥0.9. Women underwent POC-Cr testing at enrolment and 24 and 48 h. PrAKI was defined as POC-Cr >77 μmol/L, or a change ≥26 μmol/L, and staged where possible. Associations with maternal and perinatal outcomes were assessed. Multivariable logistic regression identified risk factors. Feasibility was evaluated by refusal and test failure.

**Findings:**

Of 1746 eligible women, 1669 (96%) were tested; two declined and one failed. PrAKI or chronic kidney disease occurred in 427 (25.6%). Affected women had maternal (15.4%, 66/427 versus 8.7%, 108/1239) and perinatal (19.3%, 23/119 versus 4.8%, 20/413) adverse outcomes (*p* < 0.0001). At discharge, 47%, 202/427 had unresolved kidney injury or underlying disease. Independent risk factors included age, mean arterial pressure, and pre-eclampsia or eclampsia.

**Interpretation:**

Kidney dysfunction complicates a quarter of pregnancies and is associated with adverse outcomes. POC-Cr testing may enable earlier detection and improved care in resource-limited settings.

**Funding:**

This study was funded by the 10.13039/501100000265UK Medical Research Council (MR/T038594/1) and the 10.13039/501100000272National Institute for Health and Care Research (NIHR133232).

**Research in context**

**Evidence before this study**

We searched MEDLINE, Embase, Web of Science, Scopus, Cochrane Library, Global Health, African Index Medicus, and LILACS from database inception to Aug 31, 2025, without language restrictions, and hand-searched reference lists of relevant reviews and guidelines. We combined terms for pregnancy/puerperium, acute kidney injury/renal failure/creatinine, point-of-care/near-patient testing, and low- and middle-income settings. We included randomised and non-randomised studies, prospective/retrospective cohorts, diagnostic accuracy and implementation studies in pregnant or postpartum populations; we excluded animal studies, case reports/series (<10 participants), and studies confined to non-pregnant populations. The literature shows that pregnancy-related AKI (PrAKI) remains common in LMICs with substantial maternal and perinatal morbidity and mortality, while access to timely creatinine testing is limited. Point-of-care creatinine (POC-Cr) has demonstrated analytical validity and feasibility in non-pregnant populations, but data in pregnancy are sparse and methodologically heterogeneous. A pregnancy-specific serum creatinine threshold of >77 μmol/L to flag abnormal kidney function is supported by systematic review evidence.

**Added value of this study**

This large, prospective cohort study in a low-resource tertiary maternity hospital is, to our knowledge, the first to evaluate serial finger-prick POC-Cr testing among high-risk obstetric cases. We show high feasibility (96% completion; minimal refusals/device failures), quantify a substantial burden of kidney dysfunction (25.6%), and demonstrate associations between Pr-AKI and adverse maternal and perinatal outcomes. We identify pragmatic, setting-specific operational metrics (throughput, staging where possible) and provide further evidence to support a single creatinine threshold (>77 μmol/L) with the best balance of sensitivity and specificity for predicting composite adverse outcomes, which we have validated for the first time in a LMIC context.

**Implications of all the available evidence**

Combined with prior evidence, our findings support integrating rapid creatinine assessment into obstetric triage and early-warning pathways in resource-constrained settings to enable earlier recognition of PrAKI, risk stratification, and timely management. POC-Cr could be embedded alongside validated vital-signs tools and hypertension, haemorrhage and sepsis workflows, with training and supply-chain support, and used to trigger postpartum follow-up for unresolved kidney injury or potential CKD. Priorities for research include hybrid implementation-effectiveness and cost-effectiveness studies of POC-Cr–enabled care versus standard practice, external validation of pregnancy-specific creatinine thresholds across diverse LMIC facilities, and longitudinal studies assessing maternal cardiovascular/renal sequelae and infant outcomes.

## Introduction

Pregnancy-associated Acute Kidney Injury (PrAKI) remains relatively common in low-and middle-income countries (LMICs) compared to high-income countries (HICs), with an estimated incidence of 4–26% versus 1–2.8% respectively,^1^ but estimates are limited by mainly retrospective studies with resource-dependent testing. In LMICs, associated maternal and fetal mortality are significant, ranging from 6 to 30%.^2^ Furthermore, PrAKI is now recognised as an important risk factor for chronic kidney disease (CKD), cardiovascular disease, and a fourfold increase in pre-eclampsia in subsequent pregnancies.^3^, ^4^, ^5^, ^6^ Early detection of PrAKI is essential to improve outcomes, and reduce the cost of managing long-term complications of preventable PrAKI.

Standard definitions and diagnostic criteria used for non-pregnant AKI are not validated, and likely to be limited in pregnancy^7^ given the physiological adaptations that occur: renal blood flow and glomerular filtration rate increase by 50–85% and 24–65% respectively^8^ leading to lower serum creatinine ranges compared to non-pregnant adults, and their reliance on baseline serum creatinine (not routinely measured in pregnancy), and urine output which fluctuates during pregnancy and the puerperium. This likely leads to an underestimation of the burden of PrAKI at population level, and missed or late diagnoses with resultant poor outcomes for women and their babies. As a result, serum creatinine remains the only standard, single-point assessment for kidney function in pregnancy, with a lower threshold of 77 μmol/l recommended to raise concern for PrAKI.^9^^,^^10^ This has not yet been validated in LMIC settings.

Early recognition to enable timely management significantly reduces AKI-related mortality and was highlighted by the PrAKI focused 32nd Acute Disease Quality Initiative as a research priority.^10^ Given the widespread inaccessibility of routine laboratory diagnostic tests for kidney disease, point-of-care creatinine (POC-Cr) testing offers a viable alternative. It has demonstrated validity, ease-of-use, improved efficiency and cost-effectiveness in non-pregnant patients in HICs^11^ and LMICs,^12^ with high accuracy and precision compared with enzymatic creatinine assays and iohexol-derived glomerular filtration rate (GFR).^13^ POC-Cr testing is rapid, simple, scalable and useable by non-specialist staff in both rural and referral settings, but its role in diagnosing PrAKI has not been investigated.

Sierra Leone, has one of the highest global maternal mortality rates (354/100 000)^14^ with haemorrhage, hypertensive diseases and sepsis as leading causes, with no single laboratory accredited to internationally recognised standards.^15^^,^^16^ Kidney replacement therapy (KRT) is also limited. Despite its disproportionate impact, data on PrAKI from LMICs remain limited, with no prior studies from Sierra Leone. This study aimed to describe the incidence, resolution and clinical consequences of presumed PrAKI/kidney dysfunction among pregnant and postpartum women identified as high risk by abnormal vital signs, and to assess the feasibility of serial finger-prick POC-Cr testing in a tertiary maternity hospital in Sierra Leone. We also sought to identify POC-Cr thresholds associated with severe maternal and perinatal outcomes. We prespecified hypotheses that kidney dysfunction, defined by POC-Cr >77 μmol/L and/or a KDIGO-consistent rise, would be associated with higher risk of adverse maternal and perinatal outcomes, and that hypertensive disorders of pregnancy would be associated with kidney dysfunction.

## Methods

This prospective observational cohort study was undertaken between April and August 2023 in a tertiary government maternity hospital in Freetown, Sierra Leone. Freetown is ethnically heterogeneous, with no single predominant ethnic group; Temne are the largest group alongside a historically significant Krio minority, therefore detailed race/ethnicity data were not collected and are not available for reporting. Pregnant or postpartum women with abnormal vital signs were deemed at high–high risk of PrAKI, and therefore eligible, and invited to participate. Written study information was provided, explained verbally, and participants signed or marked a consent form by thumbprint, if unable to write. Women were offered POC-Cr testing at enrolment (time 0), and at 24 and 48 h after enrolment (unless discharged sooner). Vital signs measurements (blood pressure, BP and heart rate, HR) were taken as part of routine care, with women sitting, and with the right arm supported at the level of the heart, using a pregnancy-validated device (CRADLE Vital signs alert device (VSA)^17^) which has an in-built traffic light system alerting the user to abnormal vital signs: elevated BP (systolic BP (SBP) ≥ 140 mmHg or diastolic BP (DBP) ≥ 90 mmHg) and/or shock index (SI) ≥ 0.9. The CRADLE VSA has been prospectively demonstrated to detect women at high-risk of shock secondary to postpartum haemorrhage and sepsis,^18^ two of the commonest known risk factors for PrAKI, along with hypertension.^19^ Vital signs measurements were repeated if they were abnormal on first reading, and the second reading was recorded. Normal readings were not repeated.

Following a basic 30 min training, non-clinical researchers collected 90 μl of whole blood by finger-prick and analysed the sample using the epoc® Blood Analysis System (selected as cold-chain free with high performance with NEQAS external standards,^20^ and previously validated in a non-pregnant cohort^21^) (Siemens Health Engineers USA, Tarrytown, NY), according to manufacturer’s instructions and recorded in the study database. POC-Cr results were made available to the clinical team; Pregnancy outcomes were obtained from case note review, and data quality checks were carried out by an external researcher, including adjudication by an obstetrician.

### Sample size

The incidence of PrAKI in high-risk pregnancy in LMIC has previously been estimated at 15%.^22^ With a sample size of 1,000, this study could detect an incidence of 15% with a 95% confidence interval and a precision of ±2.25% (95% CI 12.75–17.25%).

### Statistical analysis

The number of POC–Cr measurements per woman was reported and change in POC-Cr at 24 and 48 h from baseline calculated. PrAKI was defined as POC-Cr >77 μmol/l when no baseline was available, or an increase or decrease in creatinine ≥26 μmol/l. Although not validated in pregnancy, the Kidney Disease Improving Global Outcomes (KDIGO) criteria were applied to serial creatinine values (where available) to determine AKI stage.^23^ Creatinine > 77 μmol/l is a commonly used threshold in obstetric practice for identifying abnormal kidney function, identified through metanalysis,^9^ although where only one sample was available it was acknowledge that this could have also been due to pre-existing CKD.

In the absence of AKI validated definitions in pregnancy, we also sought to confirm clinical relevance of changes or thresholds of POC-Cr by exploring associations with known pregnancy complications associated with PrAKI; the pre-specified adverse maternal outcome was a composite of maternal death, eclampsia or major surgical procedure related to bleeding or infection, and additional exploratory maternal outcomes were: individual components of the composite, stroke, high dependency unit (HDU) admission, requirement for blood transfusion, dialysis, and use of magnesium sulfate. The pre-specified adverse perinatal outcome was a composite of stillbirth or neonatal death before discharge. Statistical tests used for PrAKI versus no PrAKI group comparisons were: independent groups t-test for normally distributed numeric data, median test for non-parametric numeric data, and chi-squared test for categorical data. Data were presented as mean [standard deviation, SD] for normally distributed numeric data, median [interquartile range, IQR] for non-parametric numerical data, or n (percentage, %) for categorical data.

Univariable binary logistic regression was used to evaluate the association between baseline demographic and clinical and admission characteristics and risk of developing presumed PrAKI. Statistically significant univariable factors were included in a multivariable forced entry model and area under the receiver operator curve (AUROC) was used to assess the performance of the final model. To explore whether PrAKI risk factors varied by eligibility group (high BP, or high SI) we repeated this analysis in each of these subgroups.

To determine the optimal POC-Cr threshold associated with a composite adverse maternal or perinatal outcome, test performance characteristics were calculated, including sensitivity, specificity, positive and negative predictive values (PPV and NPV), likelihood ratios (LR), and odds ratios (OR). Youden’s index was used to determine the optimal balance between sensitivity and specificity.

Statistical significance was assessed using two-tailed *p* values with *p* < 0.05 indicating statistical significance. There was no imputation for missing data. Missing values were assumed to be missing completely at random and pairwise deletion was used in the analysis.

Analysis was performed with statistical support from University of Northampton, Faculty of Health, Education and Society (SPSSv29). The study is reported in accordance with STROBE (Strengthening the Reporting of Observational studies in Epidemiology) guidelines for reporting observational studies.^24^

### Ethics statement

The study was approved by Sierra Leone Ethics and Scientific Review Committee (024/09/2022) and King’s College London Research Ethics Committee (RESCM-22/23–22669). Institutional level agreement for the study was given by Princess Christian Maternity Hospital and individual level written consent was obtained from study participants.

### Role of the funding source

The funders of the study had no role in study design, data collection, data analysis, data interpretation, or writing of the report.

## Results

**Fig. 1:**
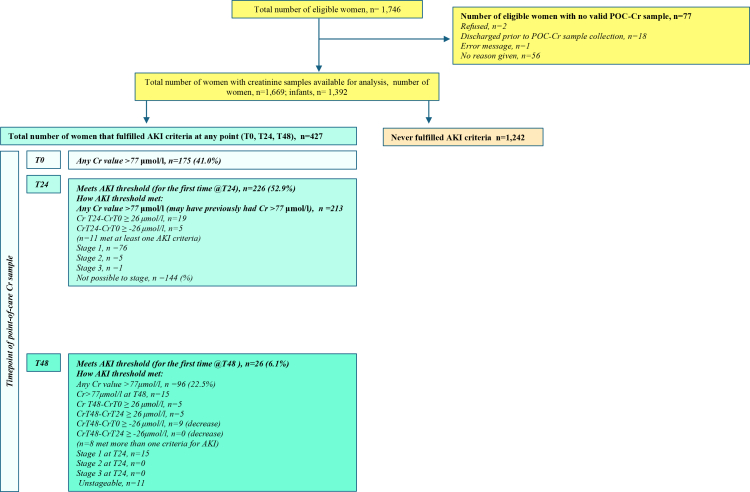
Flow diagram of participants through the study. T, timepoint; AKI, Acute Kidney Injury; POC-Cr, Point-of-care creatinine.

A three-week preparatory audit confirmed 1087/1977 (55%) of women attending maternity care met eligibility criteria. During the study period POC-Cr tests were successfully performed by non-clinical research team-members who had undergone a basic 30 min training on 1669/1746 (96%) of eligible women identified by the research team. Only two women (0.1%) refused testing, and one woman (0.06%) had no POC-Cr value available because the device failed. 769 (46.1%), 423 (25.3%) and 477 (28.6%) had one, two or three POC-Cr measurements respectively (Fig. 1). 532 (31.8%) and 1133 (67.8%) of women had their first POC-Cr test antenatally and postnatally respectively, and in five women it was not possible to determine whether the first POC-Cr test was undertaken before or after delivery. Time of discharge was recorded by the clinical team and not delayed to complete POC-Cr measurements. No adverse events were reported related to performing POC-Cr testing. Maternal demographics and admission characteristics stratified by PrAKI diagnostic criteria and KDIGO Stage, are shown in Table 1. Amongst participants, 739 (44.2%) met inclusion criteria due to high BP, and 766 (45.8%) met inclusion criteria due to high SI on CRADLE VSA. Median (IQR) haemoglobin on admission was 8 g/dl (6–10 g/dl).

**Table 1:** Maternal demographics and admission characteristics, by AKI Category, and AKI Stage as defined by Kidney Disease Improving Global Outcomes.

Demographic, Admission, Delivery, and Outcome Variables	No AKI	Any AKI	AKI (>1 AKI criteria) stage			
			Stage 1	Stage 2	Stage 3	Unstageable
Maternal demographics	n = 1239	n = 427	n = 93	n = 22	n = 10	n = 302
Age at admission, y Median (IQR)	25 (21–29)	27 (22–32)	24 (20.5–29.5)	27.5 (22–33)	22.5 (20.75–32)	27 (22–32)
Primiparous, n (%)	392 (31.6%)	100 (23.4%)	20 (21.5%)	3 (13.6%)	1 (10.0%)	76 (25.2%)
First POC-Cr test antenatally n (%)	413 (33.3%)	119 (27.9%)	19 (20.4%)	5 (22.7%)	1 (1%)	94 (31.2%)
First POC-Cr test postnatally n (%)	826 (66.7%)	307 (72.1%)	74 (79.6%)	17 (77.3%)	9 (9%)	207 (68.8%)
Admission characteristics	n = 1143	n = 384	n = 84	n = 21	n = 9	n = 270
Gestation at admission, wk Median (IQR)	36 (34–38)	36 (34–39)	36 (36–38.75)	37 (34.5–40)	37 (35.5–37.5)	36 (32.75–38.25)
High BP group, n (%)	507 (40.8%)	232 (54.3%)	46 (49.5%)	14 (63.6%)	5 (50.0%)	167 (55.3%)
‘Highest SBP’, mm Hg Median (IQR)	154 (141–170)	149 (135–167.5)	160.5 (142.8–173.5)	173.5 (146.3–180)	159 (140–175)	162 (150–180)
’Highest DBP’, mm Hg Median (IQR)	100 (93–112)	100 (90–113)	106 (100–120)	107.5 (97–114.75)	92 (78–125)	110 (100–124)
High SI group, n (%)	596 (48.0%)	170 (56.3%)	42 (51.6%)	8 (36.4%)	2 (20.0%)	118 (39.1%)
‘Lowest SBP’, mm Hg Median (IQR)	90 (80–97)	90 (80–98)	90 (75.75–97.25)	93 (82.5–94.5)	88.5 (86–88.5)	89 (80–100)
‘Lowest DBP’, mm Hg Median (IQR)	50 (43–60)	50 (43–58.25)	50 (42–55)	56.5 (47.75–59.5)	52 (50–52)	50 (43.75–60)
Nb. Below admission characteristics on ALL women						
Hb on admission (g/dl) Median (IQR)	8 (6.7–10)	8 (6–10)	8 (5.6–8)	8 (4.5–8)	7.5 (6–7.5)	8 (6–10)
Lowest Hb reported during admission (g/dl) Median (IQR)	8 (6–10)	8 (5–10)	6 (4–8)	7 (4.5–8)	7.5 (6–7.5)	8 (5.75–10)
Admission urine dipstick, n (%)						
1+ protein	30 (2.4%)	20 (4.7%)	5 (5.4%)	0 (0%)	0 (0%)	15 (5.0%)
2+ protein	45 (3.6%)	27 (6.3%)	1 (1.1%)	0 (0%)	2 (20.0%)	24 (7.9%)
3+ protein	71 (5.7%)	58 (13.6%)	14 (15.1%)	3 (13.6%)	1 (10.0%)	40 (13.2%)
Trace/negative	106 (8.5%)	40 (9.4%)	9 (9.7%)	5 (22.7%)	0 (0%)	26 (8.6%)
Missing/not done	990 (80.0%)	282 (66.0%)	64 (68.8%)	14 (63.6%)	7 (70.0%)	197 (65.2%)
Main diagnosis on admission n (%)						
Antepartum haemorrhage	79 (6.4%)	32 (7.5%)	7 (7.5%)	4 (18.2%)	1 (10.0%)	20 (6.6%)
Postpartum haemorrhage	54 (4.3%)	25 (5.9%)	5 (5.4%)	0 (0%)	1 (10%)	19 (6.3%)
Obstructed labour	108 (8.7%)	47 (11.0%)	15 (16.1%)	3 (13.6%)	1 (10%)	28 (9.3%)
Prolonged labour	33 (2.7%)	16 (3.7%)	6 (6.5%)	2 (9.1%)	0 (0%)	8 (2.6%)
Severe pre-eclampsia/eclampsia	194 (15.6%)	131 (30.7%)	29 (31.2%)	6 (27.3%)	2 (20.0%)	94 (31.1%)
Ruptured uterus	9 (0.7%)	6 (1.4%)	1 (1.1%)	0 (0%)	0 (0%)	5 (1.7%)
Puerperal Sepsis	18 (1.4%)	6 (1.4%)	1 (1.1%)	0 (0%)	0 (0%)	5 (1.7%)
Abortion complications	45 (3.6%)	9 (2.1%)	0 (0%)	0 (0%)	0 (0%)	9 (3.0%)
Ectopic pregnancy	13 (1.0%)	7 (1.6%)	1 (1.1%)	0 (0%)	0 (0%)	6 (2.0%)
Other	210 (16.9%)	59 (13.8%)	12 (12.9%)	1 (4.5%)	0 (0%)	46 (15.2%)

Results are presented as mean [SD]for normally distributed numerical data, median [IQR, interquartile range] for non-parametric numerical data or n (percentage) for categorical data. AKI indicates acute kidney injury; SBP, systolic blood pressure; DBP, diastolic blood pressure; SI, shock index; ‘High BP group’ includes women who met study eligibility criteria due to BP ≥ 140 mmHg and/or diastolic ≥90 mmHg; ‘High SI group’ includes women who met study eligibility due to SI ≥ 0.9; Hb, Haemoglobin (Hb was only available for 596 women).

A quarter of participants (427/1669; 25.6%) met PrAKI criteria during the study period. Most cases of presumed PrAKI met criteria either on admission (175; 41.0%) or at 24 h (226; 52.9%) and only 26 (6.1%) were detected at 48 h. The majority of women, 307/427 (72.1%) who met PrAKI criteria had their first POC-Cr test postnatally. Most women who met PrAKI criteria had POC-Cr > 77 μmol/l, (302, 70.7%), but did not meet KDIGO staging criteria due to single sampling (167, 39.1%) or insufficient increase/decrease in creatinine (135, 31.6%). Of those diagnosed with KDIGO criteria (125) most had Stage 1 (93, 21.8%). Overall median (IQR) change in POC-Cr between 0 and 24 h was 2.0 μmol/l (−5.0 to 9.0), and between 0 and 48 h was 5.0 μmol/l (−3.0 to 15.0).

Delivery, maternal and perinatal outcomes are shown in Table 2. During this five month study there were three maternal deaths, 143 cases of eclampsia and one stroke. No women received dialysis. The most common admission diagnoses associated with presumed PrAKI were pre-eclampsia, obstetric haemorrhage, and obstructed labour. There were 145 stillbirths and 12 neonatal deaths, including four from twin pregnancies. A significantly higher proportion of women with presumed PrAKI had composite maternal (66/427, 15.4% versus 108/1,239, 8.7%, *p* < 0.0001) and perinatal (reported per pregnancy) (23/119, 19.3% versus 20/413, 4.8%, *p* < 0.0001) outcomes compared to those without. Women with presumed PrAKI also had significantly increased rates of eclampsia, HDU admission, blood transfusions, magnesium sulfate and stillbirths than those without. We were not able to formally compare outcomes across KDIGO stages due to small numbers of women with stageable PrAKI.

**Table 2:** Delivery, maternal and perinatal outcomes, by AKI Category, and AKI Stage as defined by Kidney Disease Improving Global Outcomes.

Delivery and maternal outcomes	No AKI	Any AKI	AKI (Met at least 1 AKI criteria)			
			Stage 1	Stage 2	Stage 3	Unstageable
N	413	119	19	5	1	94
Gestation at delivery, weeks Median (IQR)	36 (34–38)	35 (30–36)	32.5 (30.25–36.75)	32 (28–32)		35 (30–36)
Twin pregnancy, n (%)	11 (2.7%)	3 (2.5%)	0 (0%)	0 (0%)	0 (0%)	3 (3.2%)
Mode of delivery, n (%)						
Caesarean section	112 (27.1%)	45 (37.8%)	12 (63.1%)	1 (20.0%)	0 (0%)	32 (34.0%)
Vaginal delivery	89 (21.5%)	38 (31.9%)	5 (26.3%)	1 (20.0%)	0 (0%)	32 (34.0%)
Missing	212 (51.3%)	36 (30.2%)	2 (10.5%)	3 (60.0%)	1 (100.0%)	30 (31.9%)
Maternal outcomes, n (%)	1239	427	93	22	10	302
Maternal composite outcome^a^	108 (8.7%)	66 (15.4%)	19 (20.4%)	3 (13.6%)	1 (10%)	43 (14.2%)
Maternal death	2 (0.2%)	1 (0.2%)	1 (1.1%)	0 (0%)	0 (0%)	0 (0%)
Eclampsia^a^	89 (7.2%)	54 (12.6%)	15 (16.1%)	3 (13.6%)	1 (10.0%)	35 (11.6%)
Major surgical procedure related to bleeding or sepsis	18 (1.4%)	11 (2.6%)	3 (3.2%)	0 (0%)	0 (0%)	8 (2.6%)
Stroke	1 (0.1%)	0 (0%)	0 (0%)	0 (0%)	0 (0%)	0 (0%)
HDU admission^a^	34 (2.7%)	35 (8.2%)	9 (9.7%)	1 (4.5%)	0 (0%)	25 (8.3%)
Blood transfusion requested^a^	281 (22.6%)	126 (29.5%)	32 (34.4%)	8 (36.4%)	3 (30.0%)	83 (27.5%)
Dialysis	0 (0%)	0 (0%)	0 (0%)	0 (0%)	0 (0%)	0 (0%)
Use of magnesium sulfate^a^	204 (16.4%)	124 (29.0%)	29 (31.2%)	5 (22.7%)	2 (20.0%)	88 (29.1%)
Perinatal outcomes, n (%) (% is per pregnancy)	413	119	19	5	1	94
Perinatal composite outcome^a^	20 (4.8%)	23 (19.3%)	2 (10.5%)	1 (20.0%)	0 (0%)	20 (21.3%)
Stillbirth^a^	18 (4.3%)	20 (16.8%)	1 (5.3%)	1 (20.0%)	0 (0%)	18 (19.1%)
Neonatal death before discharge	2 (0.5%)	3 (2.5%)	1 (5.3%)	0 (0%)	0 (0%)	2 (2.1%)

Results are presented as mean [SD] or median [IQR, interquartile range] or n (percentage). AKI, acute kidney injury; HDU, High Dependency Unit; Maternal outcome is any of: death, eclampsia or major surgical intervention related to bleeding or sepsis; Perinatal composite outcome is any of: stillbirth or neonatal death and is only available for participants recruited antenatally.

a Significant differences (*p* < 0.05) between AKI and non-AKI groups.

Table 3 shows the full results of both the univariable logistic modeling, where candidate variables were individually examined for associations with developing presumed PrAKI and the multivariable model built with only those variables showing statistically significant univariable associations with presumed PrAKI. Univariable regression found that women meeting PrAKI critiera were more likely to be multiparous, older, have a diagnosis of severe pre-eclampsia/eclampsia, and a higher mean arterial pressure (MAP) than those who did not, but after multivariable regression, only age, diagnosis of severe pre-eclampsia/eclampsia, and higher mean arterial pressure were statistically significant. For every year increase in maternal age, odds of meeting PrAKI criteria increased by 3% (OR 1.03, 95% CI 1.01–1.05, *p* < 0.0001), whilst a 1 mmHg increase in MAP increased the odds of being diagnosed with PrAKI by 2% (OR 1.02, 95% CI 1.01–1.03, *p* < 0.0001). A diagnosis of severe pre-eclampsia or eclampsia was associated with a 45% increase in the odds of meeting PrAKI criteria (OR 1.45, 95% CI 1.06–1.99, *p* = 0.020). However, performance of the multivariable model was modest, with AUROC 0.64 for predicting ≥50% association with presumed PrAKI. There was no difference in the significant variables when the univariable and multivariable logistic modeling analysis was repeated by BP group at enrolment (high BP or high SI).

**Table 3:** Results of univariable and multivariable binary logistic regression analyses to determine the association of AKI with predefined clinical, demographic and admission variables.

Predictors	Odds Ratio for a 1-unit change or Y/N for categorical variables	95% CI	Odds Ratio for 10- units change *p* < 0.05	95% CI	Model *p*-value
Univariable binary logistic regression					
Pre-admission characteristics					
History of HIV	2.41	0.99–5.86			0.052
History of Chronic hypertension	1.53	0.79–2.94			0.21
History of hypertensive disorder of pregnancy	2.03	0.93–4.40			0.074
Parity	1.09	1.01–1.18			0.034
Gravidity	0.99	0.99–1.00			0.72
Age (y)	1.04	1.02–1.06	1.43	1.18–1.71	<0.0001
Admission characteristics					
Anaemia (Hb < 9 g/dl)	1.19	0.84–1.69			0.33
Severe pre-eclampsia/eclampsia	2.31	1.79–2.99			<0.0001
Maximum MAP	1.02	1.02–1.03	1.26	1.18–1.33	<0.0001
Minimum MAP	1.05	1.01–1.02	1.57	1.07–1.27	<0.0001
Multivariable logistic regression					
Age (y)	1.03	1.01–1.05	1.29	1.06–1.55	0.010
Severe pre-eclampsia/eclampsia	1.45	1.06–1.99			0.020
Maximum MAP	1.02	1.01–1.03	1.20	1.12–1.28	<0.0001
Area under the receiver operating curve	AUROC				
	0.64				

The multivariable regression analysis was based on a forced entry model using all variables that were statistically significant predictors of AKI in the univariable analysis. CI, Confidence interval; Hb, Haemoglobin; MAP, mean arterial pressure (where MAP is calculated by diastolic blood pressure + 1/3(systolic blood pressure – diastolic blood pressure)).

First POC-Cr >80 μmol/l and >74 μmol/l were the optimal thresholds for predicting maternal and perinatal composites respectively, and the single best threshold for both outcomes was >77 μmol/l, reported in Table 4.

**Table 4:** Sensitivity, specificity, likelihood ratios, odds ratio, positive and negative predictive values calculated to determine the single creatinine threshold for the optimal balance of sensitivity and specificity for maternal and perinatal composite outcomes.

POC-Cr threshold	77 μmol/l	
	Composite maternal outcome (death, eclampsia, major surgical intervention)	Composite perinatal outcome (stillbirth, neonatal death)
Prevalence n/N, dE% (95% CI)	174/1,666, 0.10 (0.09–0.12)	43/532, 0.08 (0.06–0.12)
Sensitivity	0.34 (0.28–0.41)	0.44 (0.30–0.59)
Specificity	0.80 (0.78–0.81)	0.84 (0.80–0.87)
Positive likelihood ratio	1.68 (1.25–2.19)	4.10 (2.80–5.60)
Negative likelihood ratio	0.83 (0.73–0.93)	0.66 (0.51–0.81)
Odds ratio	2.04 (1.35–3.02)	4.10 (2.80–5.95)
Positive predictive value	0.16 (0.14–0.19)	0.19 (0.13–0.27)
Negative predictive value	0.91 (0.90–0.93)	0.95 (0.94–0.95)
AUROC	0.58 (0.54–0.63)	0.71 (0.63–0.79)

POC-Cr, Point-of-care creatinine; LR+, positive likelihood ratio; LR-, negative likelihood ratio; OR, odds ratio; PPV, positive predictive value; NPV, negative predictive value. Composite perinatal outcome is only available for participants recruited antenatally.

Overall, 47.3% (202) of women who met the criteria for PrAKI had elevated POC-Cr levels (>77 μmol/l) at their final sample before discharge (Table 5).

**Table 5:** Renal recovery at discharge.

Timepoint of test	T0 h	T24 h	T48 h	Total
n who had last POC Cr	23	200	204	427
n who had AKI at last POC-Cr	23	153	26	202
% having AKI at last POC-Cr	100%	76.5%	12.7%	47.3%

T, timepoint, POC, point-of-care; Cr, creatinine.

## Discussion

In this large prospective cohort study of high-risk pregnant women defined by abnormal vital signs in Sierra Leone, 25.5% met the criteria for PrAKI, defined by a POC-Cr measurement >77 μmol/l or according to KDIGO guidelines. Most women met criteria on admission or by 24 h, and the majority of them were postnatal at the time of their first POC-Cr test. Multivariable regression identified age, diagnosis of severe pre-eclampsia/eclampsia and MAP to be significantly associated with presumed PrAKI; each 10-year increase in age and 10 mmHg rise in MAP was associated with increased risk of meeting PrAKI criteria of 30% and 20%, respectively, whilst having pre-eclampsia or eclampsia was associated with increased risk of nearly 50%. These findings are important in the context of Sierra Leone and other low-income countries, where average total fertility rate (births per woman) remains high (Sierra Leone: 4.3) and women continue to have children later into their reproductive lifespan.^25^ In addition, in Sierra Leone and globally, hypertensive disorders of pregnancy, including pre-eclampsia, are the second leading cause of maternal death accounting for 15.8% of maternal deaths,^26^ and is the commonest admission diagnosis in the study hospital. The single optimal creatinine threshold associated with maternal and perinatal composite outcomes was >77 μmol/l, associated with high NPVs (0.91 and 0.95 respectively), the priority when the consequence of missing cases is life-threatening. PPVs (0.16 and 0.19) indicate unacceptably high risks of serious maternal and perinatal outcomes, more than justifying increased surveillance and interventions. Increasing the cut-point would improve PPV, but at the expense of worse NPV and missed diagnoses. Furthermore, a Cr threshold >77 μmol/l is aligned with recently updated recommendations to raise suspicion of abnormal kidney function in pregnancy.^9^^,^^10^ Almost half of affected women had persistently raised POC-Cr at the time of their last sample, suggesting a substantial burden of unresolved kidney injury or pre-existing CKD in the context of insufficient resource to enable repeat testing either in hospital or the community.

To our knowledge this is the largest prospective PrAKI study, and the first to use POC-Cr in pregnancy, in a low-resource setting, with serial POC-Cr samples collected from 1699 women. It provides robust incidence and outcome data in a high-risk cohort, highlighting the potential of POC-Cr to provide a more accurate epidemiological picture of the true burden of PrAKI. It demonstrates feasibility and ease-of-use (only two women refused testing, and one woman had no POC-Cr result available due to a failed test, with tests performed by non-clinical staff), and high diagnostic yield, with potential for scalability and integration of POC-Cr testing into routine care, even in community settings without laboratory support. The observed PrAKI incidence (25.5%) is high compared with previously reported rates from LMIC (4–26%)^1^ which may reflect the risk profile and complexity of the cohort as well as the use of a lower creatinine threshold of >77 μmol/l as a diagnostic criterion for PrAKI. However, this study is considerably larger than earlier studies undertaken in Africa and other LMICs with small (<50) PrAKI cohorts and diverse PrAKI definitions.^1^ Furthermore it includes all women deemed at risk, rather than only those who are able to afford creatinine testing, and demonstrates a high burden of renal impairment in young women. In this setting none of these women would have had access to creatinine testing as part of routine care due to the absence of functional in-hospital laboratory infrastructure. Extrapolating the PrAKI rate of 25.5% of this high-risk cohort over the five month study period, introduction of POC-Cr testing could enable PrAKI detection in at least 1000 women annually at the study hospital alone, with the potential for scale-up and wider impact across other hospital and community healthcare settings.

A limitation of the study is the absence of serial POC-Cr measurements: most women contributed only one or two tests, due to rapid discharge (irrespective of creatinine status), prohibiting accurate PrAKI staging and potentially underestimating disease severity. We anticipated this challenge, and therefore did not apply hierarchical models such as GEE (generalized estimated equations) or GLMM (generalized linear mixed model) to account for within-subject correlation, as is usually considered appropriate when repeated exposures are taken from the same person at different times.^27^ This approach was pre-specified and pragmatic, and consistent with the study’s objectives of evaluating feasibility, prevalence and outcome associations. Furthermore, given the high acuity of the setting, and the need for hospital beds, it would not have been appropriate to delay admission in order to complete serial POC-Cr measurements. Dialysis requirement data, a proxy measure of disease severity, were also not available, due to a lack of local access to dialysis facilities. This further highlights the need for early detection in settings where access to renal replacement therapy is limited. Another notable limitation is that we were unable to confirm the proportion of women misdiagnosed with PrAKI due to pre-existing CKD, and given the considerable burden of CKD in LMICs,^28^ it is likely that some women in this cohort were affected. The rapid discharge of many of the patients, and short follow-up could also explain the lower than expected number of maternal deaths, (n = 3), despite the large number of PrAKI cases. Access to POC-Cr, with further evaluation in studies with longer follow-up, may reveal even worse PrAKI-related outcomes, and could enable identification of strategies for earlier CKD detection that could be targeted for additional investigation and follow-up. There are concerns about POC-Cr accuracy as an alternative or adjunct to ‘gold-standard’ laboratory-based enzymatic creatinine testing. However, the less accurate Jaffe method is still used by most laboratories throughout Africa due to cost.^13^ Previously, we compared POC-Cr to NEQAS external quality standards and allowable total error (ATE) was 2%, well within desirable limits for creatinine (8%) (Unpublished data), and considerably better than no creatinine testing at all.

In keeping with other studies,^1^ hypertensive disorders, magnesium sulfate administration, transfusion (indicative of severe bleeding), and HDU admission were more frequent in women with presumed PrAKI compared to those without. Similarly, consistent with previously reported PrAKI predictors, pre-eclampsia/eclampsia and high blood pressure were found to be significantly associated with presumed PrAKI,^22^ although a model combining significant variables had modest performance, with limited clinical utility. However, unlike previous studies, in our cohort neither sepsis nor postpartum haemorrhage were associated with PrAKI, possibly related to limited diagnostics and documentation in this setting. The single POC-Cr threshold with the optimal balance between sensitivity and specificity for association with maternal and perinatal composite outcomes in this study was 77 μmol/L, consistent with the systematic review and meta-analysis of 49 studies undertaken by Wiles et al.^9^ Harel et al. subsequently proposed that even lower abnormal creatinine thresholds may be appropriate, based on a large retrospective population-based study including creatinine measurements from 243,000 pregnant women in Canada.^29^ In these studies, both Wiles and Harel demonstrate that ‘normal’ creatinine levels fluctuate with gestational age, due to pregnancy related renal adaptations, e.g.,: increase in glomerular filtration rate, with a resultant decrease in creatinine in the second trimester, followed by an increase in the third trimester. A lack of access to ultrasound scans and accurate gestational age determination limited a meaningful analysis of gestational variation in creatinine, although this is planned for future work. Furthermore, it could be argued that a single POC-Cr threshold to trigger increased monitoring and or referral is a more pragmatic and feasible approach in a high burden setting like Sierra Leone, where health care workers may have limited training. This is well-aligned with the recently published PrAKI-focused ADQI Consensus which recommends a single threshold of 77 μmol/L as a trigger for serial monitoring.^10^ However, further LMIC studies are required to establish clinically appropriate, feasible creatinine thresholds for integration into PrAKI diagnostic criteria in resource limited settings, including whether gestation-specific creatinine centiles are appropriate. Notably, 47% of women with presumed PrAKI remained above diagnostic thresholds at the time of their last POC-Cr prior to discharge, a higher proportion than reported by others in LMICs^22^ and HICs,^30^ raising concern for long-term complications, including CKD,^31^^,^^32^ cardiovascular risk,^3^ future adverse pregnancy outcomes,^6^^,^^19^^,^^31^ and adverse long-term consequences in the offspring, especially following maternal pre-eclampsia.^33^

Overall, this study reinforces the urgent need for PrAKI detection in high-risk obstetric populations. Furthermore, to prevent PrAKI, and mitigate maternal and neonatal harm, early detection of conditions commonly associated with PrAKI (e.g.,: pre-eclampsia, bleeding) should be improved where possible. A pregnancy-specific creatinine threshold >77 μmol/l to identify PrAKI or CKD could improve risk stratification and timely intervention, both ante- and postnatally; a quarter of women meeting PrAKI criteria in this study were antenatal, where knowledge of PrAKI could inform obstetric management, including timing of delivery, to reduce adverse pregnancy outcomes. Judicious early management of shock related to haemorrhage and sepsis could also be improved, two common causes of mortality in this setting. In the remaining three quarters of women, presumed PrAKI was identified after delivery, which should prompt further investigation and monitoring to moderate future risk, e.g.,: serial POC-Cr, BP and urine testing, with possible renal imaging or biopsy to identify structural damage related to Acute Kidney Disease or CKD. Given the lack of routine creatinine testing, even in tertiary centres, and the cost and logistics of external laboratory testing in low resource settings, this study highlights the clinical utility and urgent need for POC-Cr devices. An ethical concern is that if kidney injury or disease is detected, there may be limited access to appropriate care, especially if lower creatinine thresholds for PrAKI diagnosis are incorporated into clinical guidelines; however most PrAKI interventions are cheap and relatively accessible if PrAKI is detected early before complications develop. Action is underway to raise awareness, introduce PrAKI prevention into national and international guidelines and empower multi-disciplinary healthcare workers to identify and prevent PrAKI in at risk women.^34^ POC-Cr testing could also serve as an important bedside tool for earlier detection of abnormal kidney function in primary maternity healthcare settings in HICs, where increasing incidence of PrAKI is reported.^1^

Future research should focus on validating and implementing cheap, cold-chain free POC-Cr devices, in rural and primary care settings with co-development of PrAKI clinical pathways incorporating POC-Cr testing and pregnancy-specific Cr thresholds, and prospectively exploring the need for different thresholds across trimesters and postnatally, where accurate gestational age determination is possible. These pathways should be evaluated in hybrid implementation-effectiveness trials to assess clinical outcomes, cost-effectiveness, and feasibility in real-world care.

## Contributors

All authors contributed to the conception, study design and planning of the work. KK (corresponding author), RC, MM contributed to carrying out the work. KK had full access to all the raw data in the study. KK and RC verified the data. KK led the writing. All authors contributed to analysis and interpretation of the work, writing the manuscript, final approval of this version, and agree to be accountable for all aspects of the work. KK and KB had final responsibility for the decision to submit for publication.

## Data sharing statement

De-identified data is available in a repository at the following https://doi.org/10.6084/m9.figshare.29880482.

## Declaration of interests

We have no competing interests to declare.
